# Diurnal Variation of IOP in Angle Closure Disease: Are We Doing Enough?

**Published:** 2019

**Authors:** Shibal Bhartiya, Meenakshi Wadhwani, Oshin Rai, Mariah Patuel, Syril Dorairaj, Kumar Namagiri Sirish

**Affiliations:** * Glaucoma Unit Fortis Hospital, Gurugram, India; ** Chacha Nehru Bal Chikitsalya, Geeta Colony, New Delhi, India; *** Nova Southeastern University, Dr. Kiran C. Patel College of Osteopathic Medicine, USA; **** University of Florida, USA; ***** Mayo Clinic, Department of Ophthalmology, Jacksonville, Florida, USA; ****** Meenakshi Mission Hospital and Research, Madurai, India

**Keywords:** diurnal, tonometry, glaucoma, blind

## Abstract

Intraocular pressure (IOP) is known to have a definite circadian rhythm and its fluctuation correlates well with glaucoma progression. Twenty-four hour monitoring of IOP is an important indicator intraocular pressure fluctuation, as well as its peaks and spikes. However, Diurnal variation in IOP is well recognized but many decisions in glaucoma management are taken after one or two IOP measurements.

Patient directed self-tonometry can be preformed through the twenty-four cycle, and has been the subject of an ongoing debate. In this review, we studied the history of self-tonometry devices and the present technologies for future. The results of various techniques studied revealed that a standardized method of conducting diurnal variation is yet to be ascertained, and for this, a proper research method is required.

## Introduction

Defined as chronic progressive optic neuropathy, glaucoma blindness continues to maintain its reign as the second leading cause of irreversible blindness worldwide. Global measurements of disease prevalence estimate that, by 2020, the number of people with glaucoma will be around eighty million, while as many as 11.2 million will have been blinded due to glaucoma [**1**,**2**]. 

Primary glaucoma can be divided into two main categories based on angle morphology: primary open angle glaucoma (POAG) and primary angle closure glaucoma (PACG). Intraocular pressure (IOP) elevation is known to be one of the most important risk factors for both the onset and the progression of the disease in both open and angle closure glaucoma. Emerging research shows the impact of other potential modifiable risk factors, aside from IOP, that contribute to glaucoma, including socioeconomic status, nutritional intake, body mass index, obesity, exercise, smoking, and sleep apnea; many of these studies having significant limitations [**32**]. Various research studies have proven that the decrease of intraocular pressure subdues glaucoma progression, thereby preventing blindness and maintaining IOP as a modifiable risk factor.

IOP has been proven to have a definite circadian rhythm, and its fluctuation, both short term and long term, have been indicative of glaucoma progression. In fact, both peak intraocular pressure and IOP fluctuations are known independent risk factors for disease progression. Similarly, there is evidence to suggest that large diurnal intraocular pressure fluctuations may be considered as an independent risk factor for the disease [**3**-**5**]. In fact, IOP recorded during office hours is significantly less, approximately 5 mmHg, than when recorded at night [**4**]. This difference between real life conditions and the clinical setting may be more exaggerated in angle closure glaucoma eyes due to the effect that ambient lighting has on the size or dilatation of the pupil, and thus IOP [**5**-**8**]. 

Recent research has shifted its aim toward exploring diurnal variation of IOP in angle closure disease due to the importance of diurnal IOP behavior for disease management in glaucoma patients. For diurnal variation records, most of these studies have to either focus on IOP measurements during the working hours or rely on self-tonometry. There has also been some interest in 24-hour continuous measurement of IOP in angle closure glaucoma before and after iridotomy [**10**-**14**]. 

Given that angle closure disease constitutes a serious global burden of disease and blindness, this review aimed to collate the current available knowledge about diurnal variation of IOP in patients with angle closure and encourage clinicians to remember the pertinence of managing this subgroup of glaucoma patients. Keeping this in mind, an extensive search [**5**] was performed to find publications on the above subject and the 24-hour intraocular pressure monitoring devices in angle closure, to determine the various methods of studying intraocular pressure fluctuations. Cross references were also hand searched along with the expert consultation to enlarge the reference data.

## What We Know: Is Diurnal Variation In Angle Closure Disease Required? 

Some studies have shown that the risk of glaucoma progression may be higher in patients with larger fluctuations in their IOP during certain periods [**12**]. The diurnal intraocular pressure measurements in angle closure, especially in the less controlled non-clinic environment, have been intermittently documented. Few studies compared diurnal IOP fluctuation of PACG to POAG eyes, while some have looked at the IOP fluctuation through the day and correlated it with the stage of angle closure. Recently, there has been considerable interest in 24-hour continuous IOP monitoring and its relation to angle closure patients [**6**,**9**,**11**,**14**-**15**]. To better understand the physiology of IOP variations in angle closure patients, one additional diurnal IOP recording is necessary following laser peripheral iridotomy [**9**-**14**].

## Barriers To Recording Diurnal Variation In Angle Closure

The barriers for recording diurnal variation of IOP in angle closure disease are similar to those for open angle glaucoma. One of the problems with 24-hour continuous intraocular pressure monitoring is the reproducibility of the IOP recording. Predictive values of first diurnal IOP have been proven to retain low long-term reproducibility in POAG patients when evaluating the risk of IOP fluctuations. Moreover, the association of a single diurnal IOP and future IOP fluctuations is so poor that it holds limited value in clinical settings, as even between-visit agreement of IOP was poor [**33**]. In a study conducted by Bhartiya, it was found that in angle closure patients, the peak IOP was greater than office hour IOP recordings for 25% of the patients [**34**]. The many barriers to 24-hour IOP monitoring are both economically and logistically prohibitive. The cost of a sleep lab study or a continuous IOP monitor can be exorbitant and, since multiple IOP recording would be required throughout the night, normal sleep patterns would be drastically disrupted. Both researchers and clinicians have struggled to circumvent these problems [**15**,**16**].

## Strategies in diurnal IOP monitoring in Angle Closure patients

The five main strategies of 24-hour IOP monitoring that have been used are the following: 

1. Tonometry for patients using Goldmann tonometer (GAT)/ handheld tonometer.

2. Tonometry in sleep labs.

3. Self-tonometry by the patient, including Pulsair Keeler, ICare Rebound tonometer, Proview Eye Pressure Monitor (also known as phosphine self-tonometry) and Ocuton S.

4. Permanent IOP monitoring using implantable sensors.

5. Temporary IOP monitoring using contact lens sensor.

**GAT Studies**

Sihota et al. [**16**] reported a significantly higher diurnal fluctuation in eyes with open angle and angle closure glaucoma as compared to normal controls; they excluded eyes with peripheral anterior synechiae in less than three quadrants compared to normal patients, diurnal control being higher in POAG eyes treated with laser iridotomy.

Baskaran et al. found that, compared to PACS and normal patients, both PACG and PAC subjects have 2.38 times the chance of developing more than 3 mmHg of fluctuations in IOP. The study enrolled all the PACG subjects after having LPI done but before having any medical treatment [**6**].

Arora et al. [**17**] conducted a study on 100 patients with primary adult onset glaucoma (50 patients of POAG and 50 of PACG) to compare the difference between mean office hour IOP (9am to 5 pm) and diurnal IOP using GAT. The study concluded that two thirds of the patients had significant peak IOP measurements outside of office hours rather than during (p<0.003). There was a significant correlation between baseline IOP and fluctuation in IOP (r=0.61, p< 0.001). They also found that there was no difference doing IOP between 7 a.m. to 10 p.m. as compared to doing 24-hour diurnal variation. Bhartiya et al. [**15**] studied 24-hour DV in primary angle closure subjects using GAT in 77 eyes with a peripheral iridectomy done a minimum of 3 weeks prior (33 PACS, 23 PAC and 21 PACG). IOP fluctuation was reported to be significantly higher in the PACG subjects (4.4 ± 1.5 mmHg) and the PAC (5.5± 2.3 mmHg) group than the PACS subjects (4.4± 1.5mmHg). They reported a peak IOP during early morning hours at around 4 a.m. in all except for 2 eyes. Peak IOP was higher than office hour IOP in 25% of the overall subjects and in 40% of PAC/ PACG subjects. 

There are numerous studies related to Laser Iridoplasty and their effect on diurnal variation [**18**-**27**]. Of them, one of the very important studies is IMPACT study conducted by Bourne et al. [**27**], which measured DV using GAT, and compared the effect of Argon Laser peripheral trabeculoplasty (ALPI) or no ALPI in post Laser Peripheral Iridoplasty (LPI) in PACS/ PAC patients in 22 eyes. In their study, DV was done pre-ALPI and 3 months after ALPI. They concluded that diurnal IOP at 3 months was significantly reduced (5.04 mmHg ± 1.61 mmHg). Maximum IOP was significantly higher in patients without ALPI (1.87 mm Hg, p=0.026). They inferred that ALPI achieved a widening of even those parts of the angle that had remained occludable after the LPI.

In their Malmo Ocular Hypertension Study, Bengtsson et al. [**28**] conducted a RCT in 90 patients on diurnal variation using GAT to determine the effect of timolol versus placebo in untreated high IOP patients. They reported significant risk in mean IOP only and not with IOP fluctuations (p=0.49). The main limitation in their study was that they only measured three readings during office hours, whereas more readings would have led to increased accuracy. In a retrospective study that reviewed charts of 113 eyes of patients with normal tension glaucoma, Choi et al. [**29**] investigated the risk factors for optic nerve damage. None of these patients had used, or were using any anti glaucoma drugs. They evaluated the intraocular pressure over 24 hours in a hospital setting, every 2 hours from 12 p.m. to 10 a.m. the next day. During 12 a.m. and 6 a.m., IOP was measured at an interval of 3 hours. They defined fluctuation to be the difference between the highest and lowest recorded eye pressures over the duration under evaluation. They also performed a multivariate regression, and reported that neither the mean peak nor the fluctuation of intraocular pressures had a significant association with glaucomatous damage to the visual fields, or the optic nerve head. The major limitation of this study was that the authors studied the charts of the patients retrospectively. 

**Sleep Labs**

Liu et al. conducted a 24-hour sleep lab study on 33 healthy adults, to monitor the effects of posture on IOP. They regulated the lab environment, ensuring light for sixteen hours, and a dark environment for eight hours. They used a pneumotonometer to measure the eye pressures once every two hours. They divided their patients into two groups. For the first group, the eye pressure was measured with the subjects sitting in the light environment, and supine in the darkness (simulating nighttime records). For the other group, all eye pressure records were in the supine position. To maintain the environment and minimize exposure to light, the researchers wore special goggles designed for use in darkness. When measuring eye pressures during the period of darkness, the authors reported that the intraocular pressure was significantly higher during the darkness, as compared to the light period. For group one, the difference between the peak and trough IOP was 8.2+/ -1.4mm Hg. The trough was noted in the last light wake recording while the peak was recorded during the last recording in darkness. For group two, this pressure difference was found to be less, 3.8+/ -0.9mm Hg. In addition, the higher IOP recording was found in the light wake measurement. IOP measurements coincided with circadian rhythms, with the highest IOP during the end of the dark period. This indicated that the nighttime eye pressure elevation might be independent of body posture, and its changes. Moreover, even though these recordings were made in young adults without glaucoma, the trends may still be indicative of the effects of body posture in angle closure patients [**30**].

Liu et al. also evaluated the 24-hour IOP profile in 16 subjects, measuring their IOP at two hours, when sitting, and lying down in the supine position. They reported an elevation of IOP during nighttime, with daytime mean IOPs being significantly less. They also found that the IOP peaks were noted towards the end of the nighttime IOP measurements, and troughs appeared at the end of the diurnal period. The difference between the highest and the lowest eye pressures recorded was 3.8+/ -0.6mm Hg in the sitting position, and 3.4+/ -0.6 in supine position. Therefore, they concluded that there is an IOP elevation at night, in both sitting and supine positions [**31**]. 

Kida et al. evaluated the relationship between eye pressure and central corneal thickness. Fifteen young adults were evaluated during a 24-hour monitoring in a sleep lab. The eye pressure and ultrasonic pachymetry were recorded after every two hours. The highest CCT was recorded between 1:30 to 5:50 a.m., while the highest IOP was recorded at 5:30 a.m. The authors also noted that the nocturnal means were higher than the diurnal means. However, because of the difference in the time of the nighttime peaks, they did not report consistent evidence that IOP was related to CCT thickness or corneal biomechanical properties after cosine fits of the data [**32**]. 

**Self-Tonometry**

Takagi et al. conducted a study that compared eye pressure measurements using the ICare HOME Rebound Self-Tonometer with measurements from the Goldmann applanation tonometer (GAT). One hundred twenty-eight outpatient subjects diagnosed with glaucoma had their IOP measured using an ICare HOME unit by both themselves and an ophthalmologist and a GAT measurement by an ophthalmologist only. The mean GAT IOP was 12.2+/ -2.8, mean IOP using ICare by the patient (HOMEp) at home was 12.8+/ -3.7, while the mean IOP recorded by the ophthalmologist using the ICare (HOMEo) was 13.1 +/ -3.8 mm Hg, respectively. The mean difference between the HOMEp and HOMEo was found to be 0.21mm Hg (p=0.068; paired t test). Likewise, HOMEp and GAT measurement had a mean different of 0.70 mm Hg (P<0.001; paired t test), and between the HOMEo and GAT measurements the mean differed by 1.00 mm Hg (P<0.001; paired t test). Their study concluded that the ICare HOME tonometer might have a practical use for patients who wish to monitor their IOP. However, it was noted that, at times, the ICare HOME measurements were overestimated when compared to applanation tonometry. Thus, patients should use the ICare HOME tonometer for monitoring [**33**].

Asrani et al. also conducted a similar study to investigate the accuracy of the ICare IOP measurements when used by patients and trained technicians in comparison with Goldmann tonometry performed by a trained technician. They also wanted to establish if the IOP records, so obtained, were reproducible. One hundred patients had their IOP taken in the right eye by a trained technician and were then given instructions on how to take their own IOP. A different technician measured the eye pressure using GAT and was then asked to compare with the ICare IOPs previously taken by the patient and the technician. 82 patients had intraocular pressure recordings within 3 mm Hg of that recorded by the trained technician, while 75% of the recordings were within 3 mm Hg of the applanation results. Thus, authors reported that ICare rebound tonometer could be used reliably even by untrained patients. As it is generally easy to learn, this device could allow patients to self-administer and monitor their eye pressures within the comfort of their home [**34**]. 

Halkiadakis et al. also compared Icare ONE rebound self tonometer (ICRBT) with GAT. In their study, 60 patients took two readings of their eye pressure using the rebound tonometer. A trained examiner took their eye pressure using the ICRBT, and a different masked, trained examiner used the GAT. The mean difference between the rebound tonometer and GAT readings was 2.3 mm Hg (p=0.001). The IOP difference was within 3 mm for 63% of the subjects. They also found that the difference in eye pressure measurements correlated with the central corneal thickness. The IOP difference (ICRBT − GAT) was within ± 3 mm Hg for 63% of the cases. They also found that the difference in IOP measurement of ICRBT and GAT was positively correlated with CCT (r = 0.31, p = 0.015), suggesting that a greater thickness might be associated with a larger difference in IOP when using the two different appliances. Similar to the other studies, they found that the ICRBT was reliable enough in patient hands, allowing it to be used for IOP self-monitoring. They also found that ICRBT overestimated GAT measurements [**35**]. 

**Permanent Implantable IOP Sensors**

These sensors are yet to be used clinically in human beings. They may be effective in providing a continuous and true measurement of the actual intraocular pressure during all daily activities, including throughout undisturbed sleep. However, these devices come with inherent drawbacks, which have prohibited their application in clinical practice. They require an invasive surgical implantation that comes with attendant risks, including infection, bleeding, and inflammation. 

**Temporary Continuous IOP Sensor**

The Triggerfish 24-hour Contact Lens Sensor is a sensor that is embedded within a soft contact lens that records the IOP fluctuations in a subject’s eye by measuring the variations in limbal diameter. Its use has been validated in several clinical studies, and data from Triggerfish monitoring of angle closure patients has shown significant diurnal variation in eye pressures [**36**]. 

Tan et al. evaluated the differences in intraocular pressure fluctuations and their association with disease progression in 25 patients with PACG. The patients were analyzed as either glaucoma by measuring the mean deviation, classified as either having “progressive” or stable glaucoma, based on mean deviation, visual field index, and retinal nerve fiber layer thickness changes, as measured at every 6 months. They found that, in comparison with stable patients, the ones with documented progression (as ascertained by a significant change in mean deviation), had significantly different gradients (first derivative) of the intraocular pressure curves during ten to eleven p.m. and from seven to eight a.m. They also reported a significant difference in the second derivative of the intraocular pressure curve from eleven to twelve a.m., and from eight to nine a.m. Therefore, the authors concluded that 24-hour IOP data using the contact lens monitor indicated that the IOP fluctuation in patients with progressive disease is more than that observed in patients with stable glaucoma [**37**]. 

**Fig. 1:**
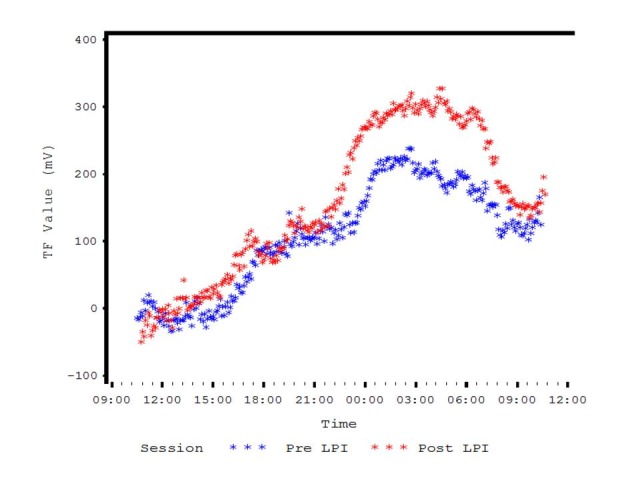
Pre and Post LPI Diurnal Variation of IOP curves in PAC

**Fig. 2:**
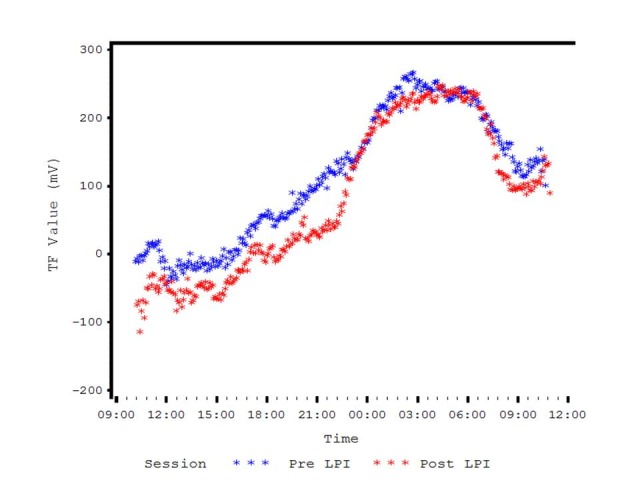
Pre and Post LPI Diurnal Variation of IOP curves in PACG

Bhartiya et al. (unpublished data, presented at AAO, 2016) evaluated the diurnal IOP curves in 12 newly diagnosed patients of PAC and PACG before and after LPI. They found that the amplitude of the acrophase was not significantly different before and after LPI. They also reported that the CLS amplitude (maximum CLS minus minimum CLS) was not significantly different when compared Pre and Post LPI. However, the difference in the mean nighttime amplitude seemed lower than the mean diurnal amplitude (**Fig. 1**,**2**). They concluded that, even though there was no change in IOP in these patients, the 24-hour IOP monitoring provided useful information regarding chronobiology of eye pressures in patients with angle closure disease [**38**] (**Fig. 1**,**2**).

Tojo et al. conducted a study using Triggerfish implantable contact lens to measure circadian IOP, twenty-four hours before and three months after phacoemulsification in patients with PACG. They measured baseline eye pressure using GAT three days before cataract surgery and concluded that there was a significant decrease in IOP from 14.7 ± 1.5 mm Hg to 11.2 ± 2.2 mm Hg at three months after cataract surgery (P=0.002). In their study, they noted that the intraocular pressure after cataract surgery in the anterior chamber anatomic parameters following cataract extraction [**39**], decreased significantly from 246±61 mVeq to 179 ± 64 mVeq. 

De Moraes et al. tested if the twenty-four hour reading of IOP measured from the CLS could correlate to the rate of visual progression of glaucoma, as evidenced from visual field loss. Forty patients who had undergone at least eight visual field tests had their mean deviation of parameters assessed through the CLS, including number of large peeks, mean peak ratio, wake to sleep slope, amplitude, and area under the cosine curve, and variability from mean. The 24-hour IOP related parameters recordings showed that the CLS was able to provide the rate of visual field progression offering a better measure of goodness of fit than Goldman parameters. Number of peaks along with mean peak ratio was their best predictor of faster progression when patients were awake. Their study also helped identify that this technology may be critical in detecting which eyes are at a higher rate of progression, during waking hours [**40**]. 

## Discussion, Implications in Disease Management and Recommendations 

Currently, patients should be made aware of the options they have, to accurately monitor their IOP to help maintain disease progressions [**41**-**43**]. To help decrease the worldwide burden of glaucoma, future studies need to focus on the mechanisms that are currently understudied or explore unknown factors other than IOP. Future research should also incorporate broader behavioral and social factors that may affect glaucoma, such as exercise, sleep apnea and the role of nutritional factors [**44**-**46**].

## References

[1] Quigley HA, Broman AT (2006). The number of people with glaucoma worldwide in 2010 and 2020. Br J Ophthalmol.

[2] Coleman A, Kodjebacheva G (2009). Risk Factors for Glaucoma Needing More Attention. The Open Ophthalmology Journal.

[3] Caprioli J, Coleman AL (2008). Intraocular pressure fluctuation a risk factor for visual field progression at low intraocular pressures in the advanced glaucoma intervention study. Ophthalmology.

[4] Hughes E, Spry P, Diamond J (2003). 24-hour monitoring of intraocular pressure in glaucoma management: a retrospective review. J Glaucoma.

[5] Tan S, Yu M, Baig N, Chan PP, Tang FY, Tham CC (2015). Circadian Intraocular Pressure Fluctuation and Disease Progression in Primary Angle Closure Glaucoma. Invest Ophthalmol Vis Sci.

[6] Baskaran M, Kumar RS, Govindasamy CV, Htoon HM, Wong CY, Perera SA, Wong TT, Aung T (2009). Diurnal intraocular pressure fluctuation and associated risk factors in eyes with angle closure. Ophthalmology.

[7] Zaretskaya RB (1948). Intraocular pressure of normal and glaucomatous eyes as affected by accessory light stimuli. Am J Ophthalmol.

[8] Lavanya R, Baskaran M, Kumar RS, Wong HT, Chew PT, Foster PJ, Friedman DS, Aung T (2012). Risk of acute angle closure and changes in intraocular pressure after pupillary dilation in Asian subjects with narrow angles. Ophthalmology.

[9] Higginbotham EJ, Gordon MO, Beiser JA, Drake MV, Bennett GR, Wilson MR, Kass MA, Ocular Hypertension Treatment Study Group (2004). The ocular hypertension treatment study: topical medication delays or prevents primary open-angle glaucoma in African American individuals. Arch Ophthalmol.

[10] Leske MC, Heijl A, Hussein M, Bengtsson B, Hyman L, Komaroff E, Early Manifest Glaucoma Trial Group (2003). Factors for glaucoma progression and the effect of treatment: the early manifest glaucoma trial. Arch Ophthalmol.

[11] Investigators AGIS (2000). Advanced Glaucoma Intervention Study (AGIS), 7. The relationship between control of intraocular pressure and visual field deterioration. Am J Ophthalmol.

[12] Gordon MO, Beiser JA, Brandt JD, Heuer DK, Higginbotham EJ, Johnson CA, Keltner JL, Miller JP, Parrish RK2nd, Wilson MR, Kass MA (2002). The Ocular Hypertension Treatment Study. Baseline factors that predict the onset of primary open-angle glaucoma. Arch Ophthalmol.

[13] Jonas JB, Budde W, Stroux A, Oberacher-Velten IM, Jünemann A (2005). Single intraocular pressure measurements and diurnal intraocular pressure profiles. Am J Ophthalmol.

[14] Barkana Y, Anis S, Liebmann J, Tello C, Ritch R (2006). Clinical utility of IOP monitoring outside of normal office hours in patients with glaucoma. Arch Ophthalmol.

[15] Bhartiya S, Ichhpujani P (2015). Diurnal Intraocular Pressure Fluctuation in Eyes with Angle-closure. J Curr Glaucoma Pract.

[16] Sihota R, Saxena R, Gogoi M, Sood A, Gulati V, Pandey RM (2005). A comparison of the circadian rhythm of intraocular pressure in primary chronic angle closure glaucoma, primary open angle glaucoma and normal eyes. Indian J Ophthalmol.

[17] Arora T, Bali SJ, Arora V, Wadhwani M, Panda A, Dada T (2015 ). Diurnal versus office hour intraocular pressure fluctuation in primary adult onset glaucoma. J Optometry.

[18] Sun X, Liang YB, Wang NL, Fan SJ, Sun LP, Li SZ, Liu WR (2010 ). Laser peripheral iridotomy with and without iridoplasty for primary angle-closure glaucoma: 1-year results of a randomized pilot study. Am J Ophthalmol.

[19] Chew PT, Yeo LM (1995 ). Argon laser iridoplasty in chronic angle closure glaucoma. Int Ophthalmol.

[20] Lai JS, Tham CC, Chua JK, Lam DS (2001 ). Immediate diode laser peripheral iridoplasty as treatment of acute attack of primary angle closure glaucoma: a preliminary study. J Glaucoma.

[21] Lam DS, Lai  JS, Tham CC, Chua JK, Poon AS (2002 ). Argon laser peripheral iridoplasty versus conventional systemic medical therapy in treatment of acute primary angle-closure glaucoma: a prospective, randomized, controlled trial. Ophthalmology.

[22] Lee JW, Lai  JS, Yick DW, Yuen CY (2013 ). Argon laser peripheral iridoplasty versus systemic intraocular pressure-lowering medications as immediate management for acute phacomorphic angle closure. Clin Ophthalmol.

[23] Ritch R, Tham  CC, Lam DS (2007 ). Argon laser peripheral iridoplasty (ALPI): an update. Surv Ophthalmol.

[24] Leung CK, Chan  WM, Ko CY, Chui SI, Woo  J, Tsang MK, Tse RK (2005 ). Visualization of anterior chamber angle dynamics using optical coherence tomography. Ophthalmology.

[25] Sng CC, Aquino  MC, Liao J, Zheng C, Ang  M, Chew PT (2016 ). Anterior segment morphology after acute primary angle closure treatment: a randomised study comparing iridoplasty and medical therapy. Br J Ophthalmol.

[26] Lai JS, Tham  CC, Chua JK, Poon AS, Chan  JC, Lam DS (2006 ). To compare argon laser peripheral iridoplasty (ALPI) against systemic medications in treatment of acute primary angle-closure: mid-term results. Eye (Lond).

[27] Bourne RRA, Zhekov  I, Pardhan S (2017 ). Temporal Ocular Coherence tomography measured changes in anterior chamber angle and diurnal intra ocular pressure after laser Iridoplasty: IMPACT Study. Br J Ophthalmol.

[28] Bentsson B, Leske  MC, Hyman L, Heiji A (2007 ). Fluctuation of intraocular pressure and glaucoma progression in Early manifest Glaucoma trial. Ophthalmology.

[29] Choi J, Kim  KH, Jeong J, Cho HS, Lee CH, Kook MS (2007 ). Circadian fluctuation of mean intraocular pressures a consistent risk factor for normal tension glaucoma. Invest Ophthalmol Vis Sci.

[30] Liu J, Kripke  D, Hoffan R, Twa MD, Loving  RT, Rex KN, Gupta N, Weinreb RN (1998 ). Nocturnal elevation of intraocular pressure in young adults. Investigative Ophthalmology & Visual Science.

[31] Liu J, Bouligny  R, Kripke D, Weinreb RN (2003 ). Nocturnal Elevation of Intraocular Pressure Is Detectable in the Sitting Position. Investigative Ophthalmology & Visual Science.

[32] Kida T, Liu J, Weinreb R (2006). Effect of 24-Hour Corneal Biomechanical Changes on Intraocular Pressure Measurement. Investigative Ophthalmology & Visual Science.

[33] Takagi D, Sawada A, Yamamoto T (2017). Evaluation of a New Rebound Self-tonometer, ICare HOME: Comparison with Goldmann Applanation Tonometer. Journal of Glaucoma.

[34] Asrani S, Chatterjee A, Wallace D (2011). Evaluation of the ICare Rebound Tonometer as a Home Intraocular Pressure Monitoring Device. Journal of Glaucoma.

[35] Halkiadakis I, Stratos A, Stergiopoulos G, Patsea E, Skouriotis S, Mitropoulos P, Papaconstantinou D, Georgopoulos G (2012). Evaluation of the ICare ONE rebound tonometer as a self measuring intraocular pressure device in normal subjects. Graefes Archive for Clinical and Experimental Ophthalmology.

[36] Agnifili L, Mastropasqua R, Frezzotti P, Fasanella I, Motolese I, Perotti E, Di Jorio A, Mattei PA, Motolese E (2015). Circadian intraocular pressure patterns in healthy subjects, rimary open angle and normal tension glaucoma patients with a contact lens sensor. Acta Ophthalmol.

[37] Tan S, Yu M, Baig N, Chan PP, Tang FY, Tham CC (2015). Circadian intraocular pressure fluctuation and disease progression in primary angle closure glaucoma. Invest Ophthalmol Vis Sci.

[38] Bhartiya S, Dada T (2016). Diurnal IOP curves in newly diagnosed patients of PAC and PACG before and after laser peripheral iridotomy. American Academy of Ophthalmology.

[39] Tojo N, Abe S, Ishida M, Yagou T, Hayashi A (2017). The fluctuation of intraocular pressure measured by a contact lens sensor in normal-tension glaucoma patients and nonglaucoma subjects. J Glaucoma.

[40] De Moraes CG, J. Jasien JV, Simon-Zoula S, Liebmann JM, Ritch R (2016). Visual Field Change and 24-Hour IOP-Related Profile with a Contact Lens Sensor in Treated glaucoma Patients. American Academy of Ophthalmology. Presented at: American Glaucoma Society Annual Meeting. February 2015, Coronado, California. J Glaucoma.

[41] Shah S, Spedding C, Bhojwani R (2000). Assessment of the diurnal variation in central corneal thickness and intraocular pressure for patients with suspected glaucoma. American Academy of Ophthalmology.

[42] Leske MC, Heijl A, Hussein M, Bengtsson B, Hyman L, Komaroff E (2003). Factors for glaucoma progression and the effect of treatment: the early manifest glaucoma trial. Arch Ophthalmol.

[43] Foster PJ, Buhrmann RR, Quigley HA, Johnson GJ (2002). The definition and classification of glaucoma in prevalence surveys. Br J Ophthalmol.

[44] Rotchford AP, Uppal S, Lakshmanan A, Lakshmanan A, King AJ (2012). Day-to-day variability in intraocular pressure in glaucoma and ocular hypertension. Br J Ophthalmol.

[45] Lam DS, Leung DY, Chiu TY, Fan DS, Cheung EY, Wong TY, Lai JS, Tham CC (2004). Pressure Phosphene Self-Tonometry: A Comparison with Goldmann Tonometry in Glaucoma Patient. Investigative Ophthalmology & Visual Science.

[46] Sultan MB, Mansberger SL, Lee PP (2009). Understanding the Importance of IOP Variables in Glaucoma: A Systematic Review. Survey of Ophthalmology.

